# Amorphous SiC/c-ZnO-Based Quasi-Lamb Mode Sensor for Liquid Environments

**DOI:** 10.3390/s17061209

**Published:** 2017-05-25

**Authors:** Cinzia Caliendo, Muhammad Hamidullah, Farouk Laidoudi

**Affiliations:** 1 Institute of Photonics and Nanotechnologies, IFN-CNR, Via Cineto Romano 42, 00156 Rome, Italy; m.hamidullah@ifn.cnr.it; 2 Research Center in Industrial Technologies CRTI, ex-CSC, P.O. Box 64, Cheraga, 16014 Algiers, Algeria; f.laidoudi@crti.dz

**Keywords:** Lamb modes, amorphous SiC, ZnO, coupling configurations, sensors, viscous liquids

## Abstract

The propagation of the quasi-Lamb modes along a-SiC/ZnO thin composite plates was modeled and analysed with the aim to design a sensor able to detect the changes in parameters of a liquid environment, such as added mass and viscosity changes. The modes propagation was modeled by numerically solving the system of coupled electro-mechanical field equations in three media. The mode shape, the power flow, the phase velocity, and the electroacoustic coupling efficiency (K^2^) of the modes were calculated, specifically addressing the design of enhanced-coupling, microwave frequency sensors for applications in probing the solid/liquid interface. Three modes were identified that have predominant longitudinal polarization, high phase velocity, and quite good K^2^: the fundamental quasi symmetric mode (qS_0_) and two higher order quasi-longitudinal modes (qL_1_ and qL_2_) with a dominantly longitudinal displacement component in one plate side. The velocity and attenuation of these modes were calculated for different liquid viscosities and added mass, and the gravimetric and viscosity sensitivities of both the phase velocity and attenuation were theoretically calculated. The present study highlights the feasibility of the a-SiC/ZnO acoustic waveguides for the development of high-frequency, integrated-circuit compatible electroacoustic devices suitable for working in a liquid environment.

## 1. Introduction

Lamb waves are acoustic modes that propagate along plates with a finite thickness: two sets of modes propagate, the symmetric and anti-symmetric modes (S*_i_* and A*_i_*, with *i* representing the mode order), whose velocities depend on the plate thickness-to-wavelength ratio [1]. Assuming as x_1_ the wave propagation direction and as x_3_ the plate normal, the symmetric modes are characterized by a longitudinal (U_1_) and transverse (U_3_) displacement components that are symmetric and anti-symmetric about the mid-plane of the plate; the anti-symmetric modes exhibit transverse (U_3_) and longitudinal (U_1_) components that are symmetric and anti-symmetric about the mid-plane of the plate. An up-to-date literature review and evaluation of ultrasonic guided waves can be found in Reference [2]. Acoustic plate modes with a displacement component normal to the plate surface are not suitable for sensing applications in liquids; when the plate contacts a liquid environment, the modes are converted into compressive waves in the surrounding liquid. The fundamental anti-symmetric Lamb mode, A_0_, while being elliptically polarized, can travel along thin plates that are in contact with a liquid, but only for a limited plate thickness range corresponding to mode velocities lower than the compressional velocity of the surrounding liquid medium. In Reference [3], the A_0_ mode propagation along amorphous SiC/cAlN composite plates is theoretically investigated, specifically addressing the design of enhanced coupling, microwave devices for liquid sensing applications. In-plane polarized modes, such as longitudinal and shear horizontal modes, are suitable for liquid sensing applications since, being the normal component of the particle displacement zero, they propagate while radiating very little energy into the liquid phase. In References [4,5,6], sensors based on the propagation of shear horizontal acoustic plate modes (SHAPMs) are described for liquid viscosity and for liquid/solid phase transition measurement applications. The fundamental symmetric Lamb mode S_0_, as well as the higher order quasi-longitudinal modes, are also suitable for liquid environment applications at specific plate thickness-to-wavelength ratios corresponding to U_3_ << U_1_. Reference [7] describes the test of a liquid sensor array based on the propagation of the fundamental and higher order Lamb modes travelling along a single crystal bulk piezoelectric material (ST-x quartz plate) and showing predominant longitudinal polarization. In Reference [8], the propagation of the fundamental S_0_ Lamb mode along thin piezoelectric membranes (BN, ZnO, InN, AlN and GaN) is theoretically studied, aiming at the design of electroacoustic devices suitable for work in a liquid environment. This study demonstrates the suitability of the thin suspended membranes for micro electromechanical systems (MEMS) applications where a high electroacoustic coupling efficiency and a high wave velocity are desirable. Moreover, unlike the single crystal bulk piezoelectric plates, this type of device can be part of an integrated system chip where the sensor coexists with the surrounding electronic circuitry. In Reference [9], some of the important characteristics of the acoustic modes propagation in both simple and composite (or multilayered) membranes are reviewed, and the terms used for the different types of modes in both the two membranes are summarized. In References [10,11], the Lamb wave propagation along bi-layered AlN/3C-SiC composite membranes is theoretically and experimentally investigated. The results clearly show that 3C-SiC-based devices can provide advantages of high frequency and high quality factor for Lamb wave devices due to the high acoustic velocity and low propagation loss. In Reference [12], some high-order quasi-Lamb wave modes in an AlN/3C-SiC plate were studied that have larger electromechanical couplings than the corresponding Lamb wave modes in an AlN thin plate. It was found that the quality factor of the third quasi-symmetric Lamb wave mode can be as high as 5510 at an operation frequency of 2.92 GHz. In Reference [13], the theoretical analysis of Rayleigh wave propagation in a-SiC/AlN and 3C-SiC/AlN demonstrates that the sensors based on a-SiC/AlN show performances that are comparable with those based on 3C-SiC/AlN, and are competitive with those based on Si(001)/AlN. An example of an a-SiC-based surface acoustic wave (SAW) device is described in Reference [14]: SAW devices implemented on a-SiC films (197 and 422 nm thick) grown on GaAs by plasma-enhanced chemical-vapor-deposition technique showed a Q in the range from 3500 to 5000, at 1 GHz operating frequency. Since the a-SiC thin film can be a useful compound for high-frequency acoustic devices, the propagation of Lamb modes in ZnO/a-SiC-based thin plates has been studied with the aim to design enhanced-coupling, microwave frequency sensors for applications in probing the solid/liquid interface. Non piezoelectric amorphous SiC substrate offers interesting properties such as a high acoustic wave velocity, resistance to chemicals, and high hardness. The a-SiC films deposition onto Si(100) substrates from a sintered SiC target by a radio frequency magnetron sputtering system has been demonstrated [13,15,16] and has the advantage of being compatible with the integrated circuit technology [17]. Piezoelectric wurtzite ZnO thin film technology has been widely used for many years for the fabrication of surface acoustic wave (SAW) devices onto non piezoelectric substrates, such as silicon, glass, and sapphire, to name just a few. When the piezoelectric ZnO film is grown onto high-velocity materials, such as diamond or SiC, it is a promising candidate for high frequency, high sensitivity micro sensors [18]. The aim of the present theoretical calculations is to investigate the influence of the thickness of both ZnO and a-SiC layers on the performances of a Lamb wave device for liquid sensing applications.

## 2. ZnO/a-SiC Plates

### 2.1. Phase Velocity Dispersion Curves

**Figure 1:**
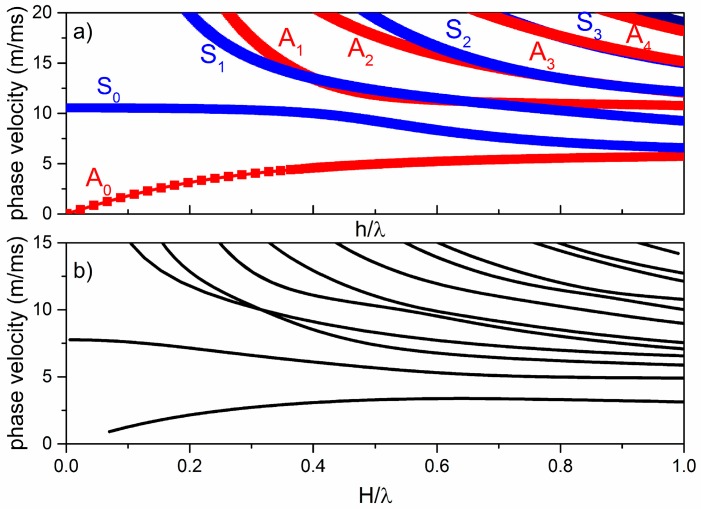
(**a**) The phase velocity vs. the plate normalized thickness h/λ of the Lamb modes propagating in a single material plate (a-SiC plate with a thickness of h = 5 µm); (**b**) The phase velocity vs. the plate normalized thickness H/λ of the Lamb-like modes propagating in a bi-layered plate with a thickness of H = 10 µm (a-SiC/ZnO plate with layers of equal thicknesses).

Lamb modes velocity dispersion curves are strongly dependent upon the material type, its crystallographic orientation, the wave propagation direction, and the plate composition (single or multilayered plates). As an example of wave propagation in homogeneous isotropic plates, Figure 1a shows the phase velocity dispersion curves of the fundamental and higher order Lamb modes propagating along the isotropic homogeneous a-SiC plate with a thickness of h = 5 µm in contact with air.

These modes are represented by two sets of curves, the symmetric (S*_i_*) and anti-symmetric (A*_i_*) modes, with *i* representing the mode order; the blue and red curves refer to the symmetric and anti-symmetric modes. For very small plate thicknesses, the S_0_ mode velocity approaches the longitudinal bulk acoustic wave velocity in a-SiC. With increasing the plate thickness, the plate becomes a half-space and the A_0_ and S_0_ mode velocities tend to approach the velocity of the Rayleigh wave in a-SiC, while the velocities of the non-fundamental modes asymptotically reach the a-SiC transverse bulk acoustic wave velocity.

Lamb-like waves propagate in a homogeneous anisotropic plate and can show three displacement components (longitudinal, shear horizontal, and shear vertical components), depending on the plate crystallographic cut and wave propagation direction. When considering a composite bi-layered plate, the symmetry of the particle displacement components with respect to the mid-plane of the plate is lost, unlike the homogeneous isotropic and anisotropic plate, and the shape of each mode changes with respect to the frequency. As mentioned in Reference [11], the fundamental modes can be considered as quasi-S_0_ and quasi-A_0_ (qS_0_ and qA_0_) for a limited plate thickness range, while all the other modes can be generically labelled as *i*th mode. Figure 1b shows the phase velocity dispersion curves of the fundamental and higher order modes propagating along a composite a-SiC/ZnO plate with a total thickness of H = 10 μm. This plate is no longer symmetric with respect to the middle plane, even if the two layers have equal thicknesses (5 µm). The two materials have different physical constants (mass density, elastic, piezoelectric and dielectric constants) and crystal symmetry, thus the mode displacement profiles are no longer simply anti-symmetric or symmetric with respect to the neutral axis. When a ZnO layer is attached to a thick a-SiC plate, the displacement profiles of the Lamb modes in the isotropic a-SiC plate are distorted due to the presence of the ZnO layer; the phase velocity dispersion curves of the composite plate, shown in Figure 1b, have equal color as the distinction between mode types is somewhat artificial. The theoretical phase velocity dispersion curves were calculated utilizing the ZnO and a-SiC material constants available in the literature [15,16,19] and using the COMSOL, DISPERSE [20], and MATLAB software.

### 2.2. Displacement Profile

The shape of the modes travelling in a bi-layered plate are no longer simply anti-symmetric or symmetric with respect to the neutral axis, but are distorted. The mode shape at different points along the same dispersion curve evolves continuously from symmetric to anti-symmetric and vice versa, thus it would be incorrect to label the dispersion curves as quasi symmetric or quasi anti-symmetric. We decided to assign each dispersion curve a number in the order in which they appear along the frequency axis. We investigated the fundamental quasi symmetric mode and two higher order modes that show, for specific thickness-to-wavelength ratio values, a predominant longitudinal polarization (U_3_ << U_1_) on the a-SiC layer-free side of the plate. These modes were studied with the aim to design a high frequency electroacoustic device suitable to work in a liquid environment. The latter modes are hereafter named quasi longitudinal modes, qL_1_ and qL_2_.

#### 2.2.1. The qS_0_ Mode

**Figure 2:**
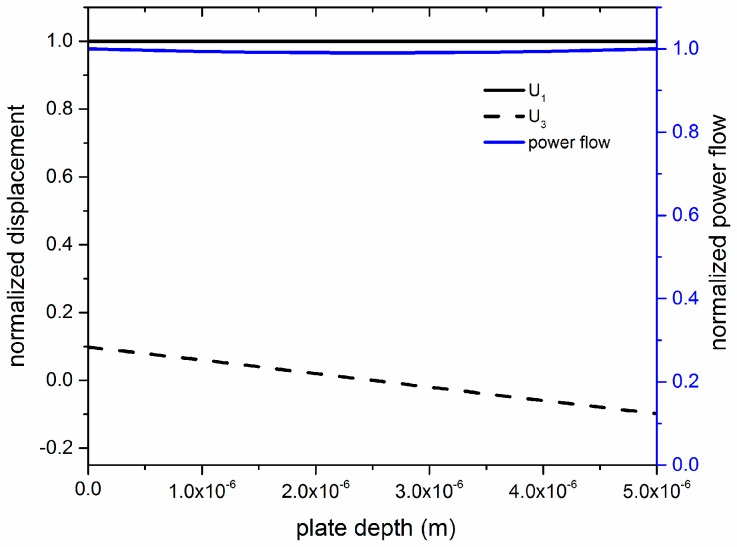
The U_1_, U_3_ and power flow depth profile for an a-SiC plate with a thickness of 5 µm (h_aSiC_/λ = 0.1, λ = 50 µm).

The investigation of the acoustic field profile across the thickness of the a-SiC bare plate revealed that, up to an a-SiC thickness-to-wavelength ratio equal to h_aSiC_/λ = 0.1, the S_0_ mode has polarization predominantly oriented along the propagation direction. Figure 2 shows the U_1_, U_3_ and power flow depth profile for an a-SiC plate with a thickness of 5 µm (h_aSiC_/λ = 0.1, λ = 50 µm). The wave travels at velocity v = 10,522 m/s. U_1_ and U_3_ have been normalized to the U_1_ value at the a-SiC plate surface; the power flow has been normalized to its value at the a-SiC plate surface.

**Figure 3:**
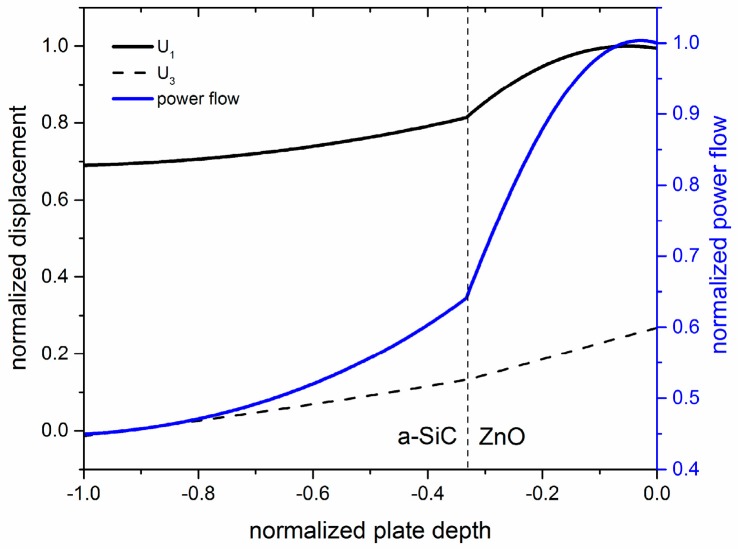
The power flow, and the longitudinal and shear vertical particle displacement components, U_1_ and U_3_, of the qS_0_ mode propagating along an a-SiC plate with h_aSiC_/λ = 0.1, covered by a ZnO layer with h_ZnO_/λ = 0.05.

When a thin ZnO layer is added to the a-SiC plate, the qS_0_ mode field profile results quite unperturbed for a very small ZnO thickness range. The power transported by the mode along the plate per unit length perpendicular to the propagation direction and per unit time, Pn=12ρω2vgrn∫−h2h2(|U1| 2+ |U3| 2)dx3, was calculated for waves propagating along the x_1_ direction of the plate, with U_1_ and U_3_ being the longitudinal and transverse particle displacement components normalized to the power flow evaluated at the x_3_ = h/2 surface; ω = 2π·f is the angular frequency, f = v_ph_/λ, ρ is the plate mass density, n is the mode order, and v_gr_ is the group velocity of the mode. The *P* distribution inside the plate gives information about the location of the peaks of acoustic energy transmission. Figure 3 shows the qS_0_ mode field profile and power flow distribution into the plate depth for a-SiC and ZnO normalized thicknesses equal to 0.1 and 0.05 (λ = 50 µm), respectively; the mode travels at velocity v = 8293 m/s. As can be seen, the longitudinal displacement component U_1_ is almost constant across the composite plate depth, while U_3_ is negligible on the a-SiC side of the plate, and it increases slowly up to the ZnO side where it reaches a non-negligible value.

#### 2.2.2. The qL_1_ and qL_2_ Modes

**Figure 4:**
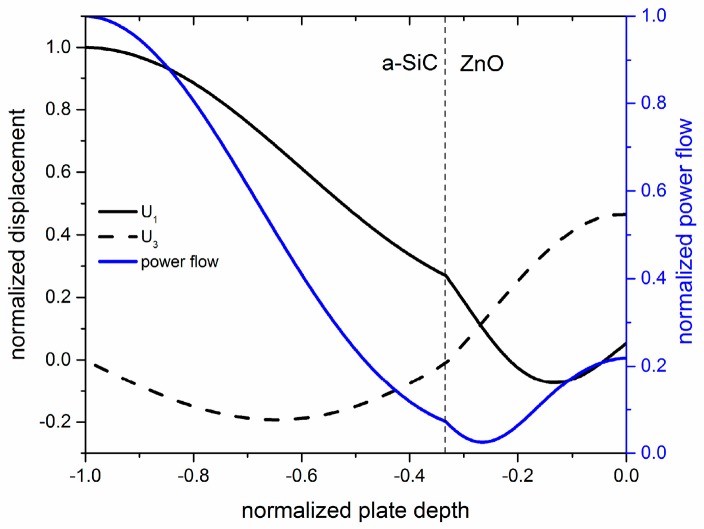
The field profile and in-plane power flow per unit area for the qL_1_ mode propagating along a-SiC and ZnO thicknesses equal to 5 and 2.5 µm, for λ = 16.7 µm.

**Figure 5:**
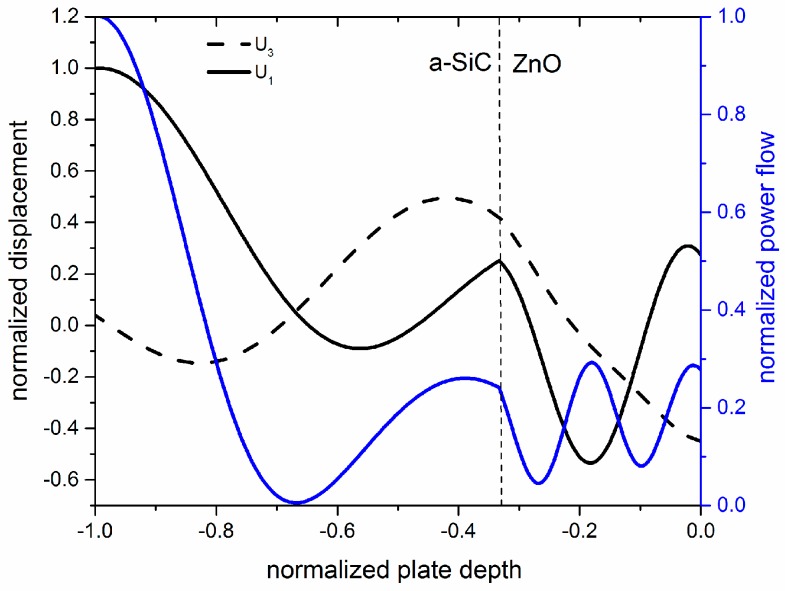
The field profile and in-plane power flow per unit area for the qL_2_ mode propagating along a-SiC and ZnO thicknesses equal to 5 and 2.5 µm, for λ = 8.8 µm.

As an example, Figure 4 and Figure 5 show the field profile and the in-plane power flow per unit area for the modes named quasi-longitudinal modes, qL_1_ and qL_2_, propagating at a velocity equal to 11,456 and 12,346 m/s, respectively, along the composite plate with a-SiC and ZnO fixed thicknesses (5 and 2.5 µm), for λ = 16.7 and 8.8 µm. The displacement components as well as the power flow have been normalized to their maximum values. As can be seen, these two modes have a very small U_3_ component at the a-SiC-free surface where U_1_, as well as the power flow, are at maximum values, thus confirming that these modes are suitable for the development of electroacoustic devices for applications to a liquid environment.

**Figure 6:**
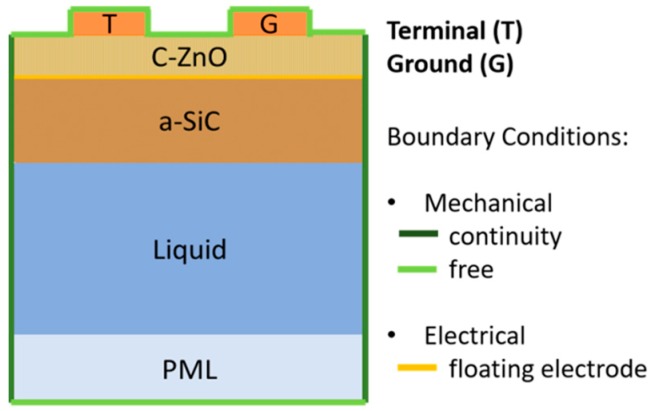
The FEM schematic.

**Figure 7:**
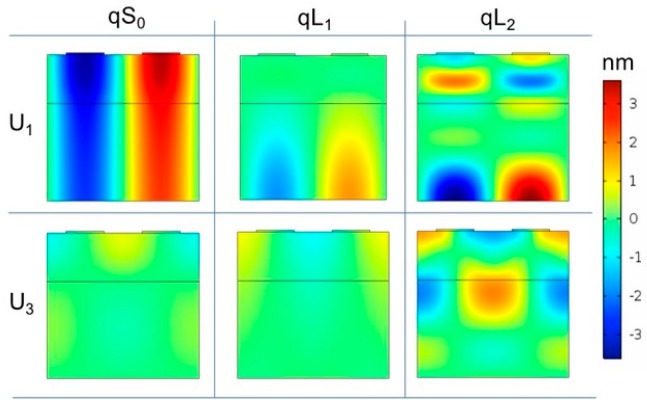
The U_1_ and U_3_ components of the three modes travelling along the composite plate in air with a-SiC and ZnO fixed thicknesses (5 and 2.5 µm), the plate width equal to one λ, and λ = 50, 16.7, and 8.8 µm for qS_0_, qL_1_, and qL_2_, respectively.

COMSOL FEM Multiphysics software was employed to simulate the resonance mode shape of the three modes, qS_0_, qL_1_, and qL_2_, propagating in the a-SiC/ZnO composite plate, with and without the liquid contacting the plate surface. 2D piezoelectric device simulation with solid mechanics and electrostatic modules was used for eigen-frequency analysis of the composite plate. The number of degrees of freedom to solve for the mode is minimized by providing periodic boundary conditions to the transmitting interdigital transducer which is a one-finger structure with a total width of one wavelength. Figure 6 shows the schematic of the COMSOL model with boundary conditions; as the liquid was modeled as a half space layer, a perfectly matched layer (PML) was added so that the wave propagating in the liquid was not reflected back to the plate. Figure 7 shows the U_1_ and U_3_ components of the three modes travelling along the composite plates in air; the a-SiC and ZnO thicknesses are fixed (5 and 2.5 µm), and the wavelength is λ = 50, 16.7 and 8.8 µm for the three modes.

**Figure 8:**
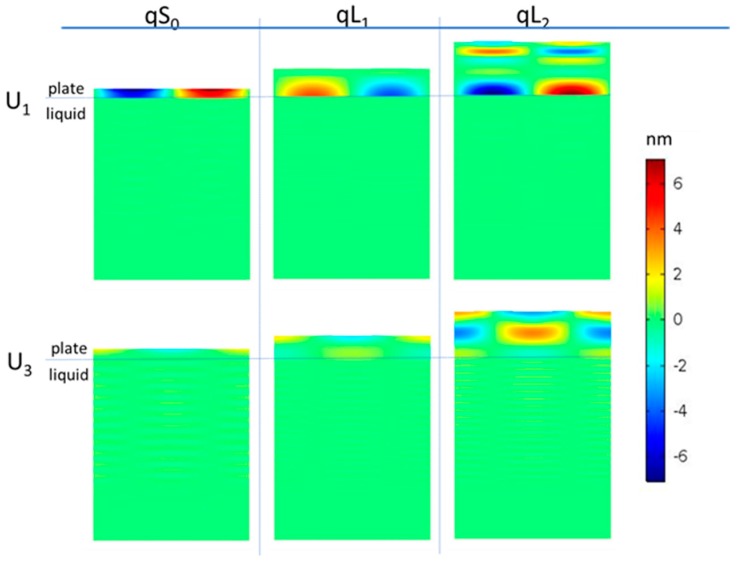
The displacement components U_1_ and U_3_ of the three modes propagating along the a-SiC/ZnO plate that makes contact with the liquid. The a-Sic and ZnO layers have a thickness equal to 5 and 2.5 μm; the plate width is equal to one λ; the H/λ = 0.15, 0.45, and 0.85, for qS_0_, qL_1_, and qL_2_, respectively.

Figure 8 shows the displacement components U_1_ and U_3_ of the three modes propagating along the a-SiC/ZnO plate that contacts the liquid environment from the a-SiC side of the plate. As can be seen, the acoustic energy is confined inside the plate. The liquid was modelled as a linear isotropic viscoelastic material with independent elastic constants, the bulk (K) and shear (G) moduli, and two independent bulk (η_b_) and shear viscosities (η_v_) from Reference [21].

#### 2.2.3. The Coupling Coefficient Dispersion Curves

**Figure 9:**
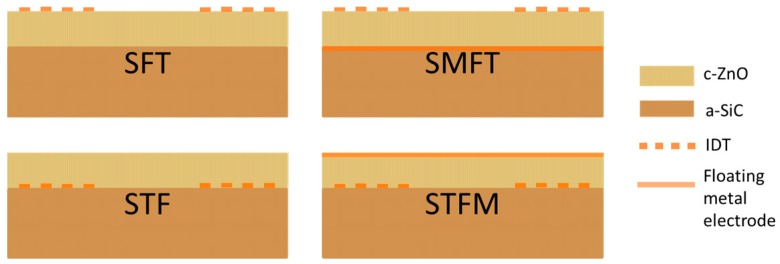
Cross-sectional illustrations of the four coupling configurations on a composite membrane including a piezoelectric layer (Film) onto a non-piezoelectric layer (Substrate): substrate/film/transducer (SFT), substrate/metal electrode/film/transducer (SMFT), substrate/transducer/film (STF), and substrate/transducer/film/metal electrode (STFM).

Lamb mode devices based on piezoelectric layers use input and output interdigital transducers (IDTs) to launch and receive the acoustic waves. In the a-SiC/ZnO composite plate, four piezoelectric coupling configurations can be obtained by placing the IDTs at the a-SiC/ZnO interface (STF) or at the ZnO-free film surface (SFT), further including a floating electrode opposite the IDTs (STFM and SMFT). The four configurations are depicted in Figure 9.

**Figure 10:**
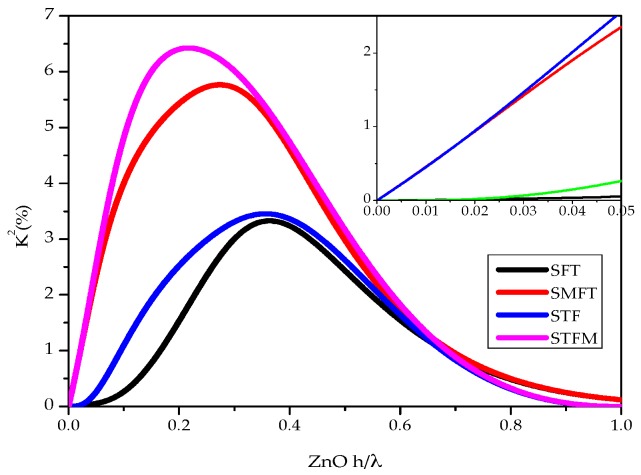
The K^2^ vs. the ZnO thickness-to-wavelength ratio for the four coupling configurations. The a-SiC normalized thickness is fixed at h/λ = 0.1.

The electromechanical coupling coefficient K^2^ is an important parameter used as a direct estimate of the electrical to acoustic energy conversion efficiency; it is frequency dispersive and strongly affected by the electrical boundary conditions that influence the potential depth profile. K^2^ can be approximated as K2=2·(vf−vm)vf , where v_f_ and v_m_ are the velocities along the electrically open and shorted surfaces of the ZnO film. The K^2^ of the qS_0_ mode was studied with respect to the thickness of the a-SiC and ZnO layers and the electrical boundary conditions. The K^2^ dispersion curves of the four configurations are shown in Figure 10: the a-SiC normalized thickness is fixed (h_aSiC_/λ = 0.1); the abscissa of the graph represents the ZnO normalized thickness. As can be seen, the K^2^ curves exhibit a large dependence on the IDTs and floating electrode arrangement [22]. The K^2^ of the qS_0_ mode in the STFM coupling configuration is larger than that of the other configurations; the qS_0_ mode-based devices can reach remarkable K^2^ values, from 3.3% for the SFT configuration up to about 6.5% for the STFM configuration.

While designing a qS_0_ mode-based device suitable for contact with a liquid environment, the choice of the ZnO layer normalized thickness is restricted to the small range shown in the inset of Figure 10: it follows that the SMFT and STFM coupling configurations must be preferred to the SFT and STF configurations for their higher K^2^. Table 1 lists the K^2^ of the four coupling configurations based on the propagation of the three modes, qS_0_, qL_1_, and qL_2_. The STFM configuration is the most efficient for the qS_0_ and qL_1_ modes, while the SMFT is the most efficient for the qL_2_ mode. The K^2^ of the selected configuration is a critical factor to determine the optimum number of electrode finger pairs, Nopt=π4K2, of both the input and output IDTs, required to obtain the minimum insertion loss and the maximum frequency bandwidth of the filter or delay line to be implemented on the a-SiC/ZnO plate.

## 3. Lamb Wave Sensors

### 3.1. Gravimetric Sensor

The most common sensing application for electroacoustic devices is based on the gravimetric principle for mass detection. A mass accumulation on the device surface changes the surface density of the propagating medium, resulting in a mode velocity shift. If the added mass consists of an ideal thin elastic film that moves synchronously with the oscillating surface, the fractional velocity change to the added mass ratio defines the gravimetric sensitivity of the phase velocity Smv= (Δvv0)m, where m = ρ·h_am_, ρ and h_am_ are the added layer’s mass density and thickness, and *v* and *v*_0_ are the unloaded and mass-loaded plate's phase velocity. If the gravimetric detection occurs inside a liquid environment, then the wave attenuation is supposed to also be affected by the liquid environment’s characteristics. The gravimetric sensitivity of the Insertion Loss (IL), SmIL=ΔILm, was calculated by IL=−40π(log10e)vivr= −54.6 vivr, where v_r_ and v_i_ are the real and imaginary parts of the wave velocity, $\Delta \mathrm{IL}={\mathrm{IL}}_{0}-{\;\mathrm{IL}}_{m}$, and IL_0_ and IL_m_ are the insertion loss without and with the added mass attached to the sensor surface. The sensitivity calculation was performed for two different liquid environments, water and a 53% glycerol/water mixture, to estimate the effect of the liquid environment’s viscosity on the sensor performance. It was assumed that the added mass is anchored onto the a-SiC side of the composite plate. The velocity and loss sensitivities to the added mass in liquid were calculated for the qS_0_, qL_1_, and qL_2_ modes travelling in the a-SiC(5 µm)/ZnO(2.5 µm) plate, with λ equal to 50, 16.7, and 8.8 µm, respectively. The relative velocity changes per unit added mass in water are equal to −109, −488, and −628 cm^2^/g, for the qS_0_, qL_1_, and qL_2_ modes, respectively. In the 53% glycerol/water mixture, the calculated sensitivities were equal to −110, −511, and −685 cm^2^/g for the qS_0_, qL_1_, and qL_2_ modes, respectively. The velocity gravimetric sensitivity of the qS_0_ mode was scarcely affected (about 1% enhancement) by the viscosity of the liquid environment, while for the qL_1_ and qL_2_ modes an increase of about 5% and 9% was observed. The IL changes per unit added mass in water were equal to 0.54 × 10^−3^, 13.7 × 10^−3^, and 38.4 × 10^−3^ dB/λ mm^2^/μg for the qS_0_, qL_1_, and qL_2_ modes. The IL changes per unit added mass in the 53% glycerol/water mixture resulted in about 16 times that observed in water: it was equal to 10 × 10^−3^, 176 × 10^−3^, and 553 × 10^−^^3^ dB/λ mm^2^/μgr for the qS_0_, qL_1_, and qL_2_ modes.

A Lamb wave delay line consisting of two IDTs with fingers width and spacing equal to λ/4, with a number of finger pairs Nopt=π4Kmax2 equal to 5, 10, and 20 (being $K_{\max}^{2}$ the highest K^2^ value between those listed in Table 1 for each mode), and with a center-to-center IDT spacing of 3N_opt_·λ could experience an IL equal to about 8 × 10^−3^, 0.4, and 2.3 dB mm^2^/μgr in water, and 0.15, 5.3, and 33 dB mm^2^/μgr in the mixture of 53% glycerol in water. For many liquid media sensing applications, a mass anchored to the sensor surface has to be detected that is not necessarily well-represented by a continuum layer rigidly coupled to the device surface, as assumed here. Thus, the calculated mass sensitivity in liquid media should be understood as a purely qualitative tool for comparisons between acoustic wave sensors, and yields only limited information regarding the response that could be observed in a real experiment [23].

### 3.2. Viscosity Sensor

**Figure 11:**
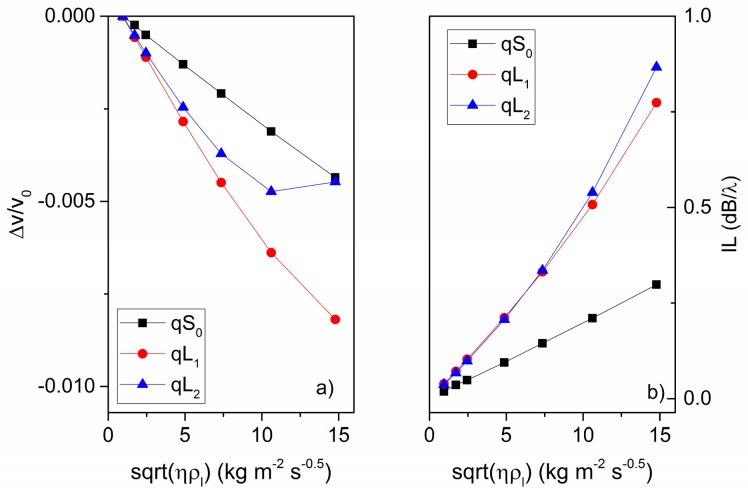
(**a**) The wave relative velocity change and (**b**) the IL vs. the $\sqrt{{\eta \rho }_{l}}$, where η and ρ_l_ are the viscosity and density of a water/glycerol mixture contacting the a-SiC side of the composite plate.

When a liquid contacts the acoustic waveguide, the in-plane particle displacement component of the acoustic mode couples to a very thin viscous boundary layer of thickness δ=2ηωρl, where η and $\rho_{l}$ are the liquid viscosity and mass density. The viscous liquid was supposed to be a mixture of water and glycerol; the fraction of glycerol by volume ranged from 0 (only water) to 0.6, and the $\sqrt{{\eta \rho }_{l}}$ ranged from 0.95 to about 15 kg·m^−2^·s^−0.5^ [21]. The effects of both the viscosity η and the mass density $\rho_{l}$ of the water/glycerol mixture on the wave velocity and IL was analyzed numerically. The real and imaginary parts of the phase velocity (v_i_ and v_r_) of the three modes were calculated for different concentrations of the water/glycerol mixtures. The relative changes of the phase velocity Δv/v_0_ and the IL as a function of $\sqrt{{\eta \rho }_{l}}$ are shown in Figure 11a,b. It was assumed that the examined glycerol-water mixture was in contact with the a-SiC surface of the composite plate. 

As shown in Figure 11, at low values of viscosity, the fluid behaves as a Newtonian liquid with Δv/v_0_ and IL proportional to $\sqrt{\rho_{l}\eta }$. This is observed for the qS_0_ and qL_1_ modes, whose time scale (wave period equal to about 6 and 1.5 ns) is far larger than the fluid relaxation time τ = η/μ (where μ the liquid shear modulus) for the $\sqrt{\rho_{l}\eta }$ = 0.9 to 15 abscissa range. The relative velocity shift of the qL_2_ mode is linearly dependent on $\sqrt{\rho_{l}\eta }$ for low viscosity values, and is reversed for glycerol/water percentage ≥44%, as τ becomes close to the wave period (0.8 ns) of the mode [24,25]. The relative resonant frequency shift and the IL shift per unit change in the square root of the density-viscosity product are equal to (Δff0)/ρlη = −316 ppm·m^2^·s^0.5^·kg^−1^ and $\mathrm{IL}/\sqrt{\rho_{l}\eta }$ = 0.02 dB/λ·m^2^·s^0.5^·kgr^−1^ over the range of 0 to 60% glycerol in water, for the qS_0_ mode; (Δff0)/ρlη = −702 ppm m^2^·s^0.5^·kgr^−1^ and $\mathrm{IL}/\sqrt{\rho_{l}\eta }$ = 0.045 dB/λ for the qL_1_ mode, and (Δff0)/ρlη = −579 ppm·m^2^·s^0.5^·kgr^−1^ and $\mathrm{IL}/\sqrt{\rho_{l}\eta }$ = 0.046 dB/λ·m^2^·s^0.5^·kgr^−1^ for the qL_2_ mode, in the low viscosity region from 0 to 44% glycerol in water. For a Lamb wave delay line with two IDTs with N_opt_ finger pairs, 3N_opt_·λ IDTs center-to-center distance, a unit change in the square root of the density-viscosity product will produce an IL increase equal to 0.3, 1.35, and 2.76 dB·m^2^·s^0.5^·kg^−1^.

## 4. Conclusions

The propagation of quasi Lamb modes along a-SiC/c-ZnO composite plates have been investigated by theoretical calculation with respect to the c-axis oriented ZnO and amorphous SiC films’ thicknesses and electrical boundary conditions. The displacement profiles, phase velocities, and electromechanical coupling coefficients of four ZnO/SiC-based coupling configurations have been theoretically studied, specifically addressing the design of enhanced-coupling, microwave frequency sensors for liquid environments. The IL and velocity changes of the qS_0_, qL_1_, and qL_2_ modes when contacting a viscous Newtonian liquid have been calculated for different viscosities and added mass values. The phase velocity and loss sensitivities to the added mass and viscosity have been theoretically studied for the qS_0_, qL_1_, and qL_2_ modes in the a-SiC/ZnO plates. The ZnO/SiC-based sensors are proven to achieve remarkable performances (high sensitivity and enhanced coupling efficiency) that are important prerequisites for the design of future devices to be used in the context of chemical, biological, and physical quantities detection.

## Figures and Tables

**Table 1:** The K^2^ of the qS_0_, qL_1_, and qL_2_ modes travelling in the a-SiC (5 µm)/ZnO (2.5 µm) plate, for λ = 50, 16.7, and 8.8 µm.

Mode /K^2^ (%)	Coupling Configuration			
	SFT	SMFT	STF	STFM
qS_0_	0.054	2.35	0.26	2.56
qL_1_	0.08	0.54	0.33	0.79
qL_2_	0.15	0.20	0.084	0.13
